# Familiarity with Speech Affects Cortical Processing of Auditory Distance Cues and Increases Acuity

**DOI:** 10.1371/journal.pone.0041025

**Published:** 2012-07-20

**Authors:** Matthew G. Wisniewski, Eduardo Mercado, Klaus Gramann, Scott Makeig

**Affiliations:** 1 Department of Psychology, University at Buffalo, The State University of New York, Buffalo, New York, United States of America; 2 Swartz Center for Computational Neuroscience, Institute for Neural Computation, University of California San Diego, La Jolla, California, United States of America; 3 Institute for Cognitive Science, University of Osnabrück, Osnabrück, Germany; Max Planck Institute for Human Cognitive and Brain Sciences, Germany

## Abstract

Several acoustic cues contribute to auditory distance estimation. Nonacoustic cues, including familiarity, may also play a role. We tested participants’ ability to distinguish the distances of acoustically similar sounds that differed in familiarity. Participants were better able to judge the distances of familiar sounds. Electroencephalographic (EEG) recordings collected while participants performed this auditory distance judgment task revealed that several cortical regions responded in different ways depending on sound familiarity. Surprisingly, these differences were observed in auditory cortical regions as well as other cortical regions distributed throughout both hemispheres. These data suggest that learning about subtle, distance-dependent variations in complex speech sounds involves processing in a broad cortical network that contributes both to speech recognition and to how spatial information is extracted from speech.

## Introduction

An important property of auditory perception is the ability to locate the origin of a sound. One fundamental question about this ability is, ‘How do listeners process auditory inputs to estimate their distance from a sound source?’ Early attempts to answer this question focused on the physical characteristics of received sounds that might provide distance cues. The most prominent cue is sound intensity; sounds coming from nearby are generally louder than ones that have traveled long distances [1]. The spectral content of a sound can also provide relevant cues: during propagation in air, high frequencies attenuate more rapidly than low frequencies [2]. The effort exerted in communicating speech over long distances can also change the spectral information within speech sounds [3], [4]. In addition, the direct-to-reverberant energy ratio (energy reaching the listener directly versus via reflecting surfaces) decreases with increasing source distance, providing yet another acoustic cue for distance estimation [2], [5].

Despite the abundance of physical cues that potentially could provide information about distance to a sound source, humans are generally poor at making auditory distance judgments [3], [6], [7]. One common finding is that people tend to underestimate the distances of far sound sources and to overestimate the distances of nearby sources [2], [3]. Furthermore, thresholds for detecting changes in the distance of an auditory sound source generally correspond to about a 13% change in distance and can be as much as 48% for nearby sounds 7,8. This is much higher than thresholds for detecting differences in horizontal azimuth, which can be near 1% for frontal sound sources [8], [9].

The physical characteristics of sounds are not the only factors that determine how well a listener can judge auditory distance. If familiar sounds are used to test distance perception, performance is better than if unfamiliar sounds are used [3], [6], [10]. In one study, forward and backward speech sounds were recorded at distances of 2 m and 30 m [10]. Participants were played pairs of these sounds and were asked to indicate whether the source of the second sound of the pair was closer, further, or equidistant from the first. In this task, distance perception of familiar stimuli (forward speech) was significantly above chance, whereas distance perception of unfamiliar stimuli (backwards speech) did not differ from chance. Similarly, Brungart and Scott [3] tested the ability of participants to estimate the distances of forward and backwards speech sound sources that were recorded at distances of.5, 1, 2, 4, 8, 16, 32, 64, and 128 m in a large open field. When speech sounds were played backwards, there was a substantial decrease in accuracy relative to versions of the same speech samples played forward. Because backwards speech had identical spectral information as normal speech, Brungart and Scott [3] suggested that listeners use both acoustic and phonetic information in order to make accurate distance judgments. However, both of these studies confounded phonetic familiarity with word familiarity. For instance, the speech sequence “Don’t ask me to carry an oily rag like that” (a sequence used in [3]) contains both familiar words and familiar phonemes, leaving it unclear which type of familiarity might facilitate performance.

To our knowledge, no studies have attempted to look at the neural mechanisms underlying familiarity effects on auditory distance perception in humans, and very few have examined the neural substrates that underlie processing of physical auditory distance cues (for review see [8], [11]). The few researchers studying neural correlates of auditory distance perception have looked at cortical areas associated with auditory processing. In one magnetoencephalographic (MEG) study, streams of white noise bursts with duration and amplitude deviants were presented to human participants [12]. Auditory evoked magnetic fields measured over the left and right supratemporal planes revealed that amplitude deviants led to a larger evoked response over the right supratemporal plane than over the left. Given that amplitude can provide a cue to distance, the authors speculated that the right temporal lobe is important for detecting the distance of a sound source. Right temporal areas have also been implicated in the tracking of changes in auditory distance in a functional magnetic resonance imaging (fMRI) study in which sounds that increased in amplitude over time activated a distributed network of brain regions including the right temporal/parietal junction, right motor and pre-motor areas, parts of cerebellar cortex, and the midbrain [13]. These findings suggest that non-auditory areas may also be important for judging sound source distance.

In the current study we attempted to explore differences in participants’ ability to make auditory distance judgments between sounds with similar physical characteristics, but having different levels of phonetic and lexical familiarity. We used sounds that were lexically and phonetically familiar (English speech), only phonetically familiar (Bengali Speech), or both lexically and phonetically unfamiliar (backwards English and Bengali Speech). We also intensity-normalized all sounds to investigate the neural mechanisms underlying the perception of distance without intensity cues, as there appears to be little neuroimaging work investigating other cues available for auditory distance perception. We hypothesized that participants would be better at distinguishing near and far sources of familiar sounds. In addition, if lexical familiarity aids auditory distance perception, then source distance for English speech should be more distinguishable for native English speakers, whereas if phonetic similarity is sufficient, then there should be no advantage for English speech over Bengali speech.

We used high-resolution EEG processed by independent component analysis (ICA) to investigate brain processes that underlie differences in the judgment of distance for intensity-normalized speech sounds varying in familiarity. EEG scalp signals are each mixtures of potentials volume-conducted from cortical brain processes plus non-brain artifact processes (eye movements, scalp and neck muscle activities, electrocardiographic signals, line noise, etc.). Using ICA, multi-channel EEG signals can be separated into independent component (IC) processes that can be attributed to particular brain regions or non-brain origins [14], [15]. This approach provides a powerful tool for identifying activities in brain regions of interest, as well as sufficient temporal and spatial resolution to index the distributed network of brain areas whose joint activities may underlie auditory distance judgment [13] including familiarity effects.

Since speech familiarity has been shown to facilitate distance perception [3], [6], [10], we expected that many of the same cortical regions involved in speech recognition (e.g., left temporal and frontal regions [16]) might also contribute to judgments of the distance from a speaker. Given the difficulty of the task, we also expected to find changes in EEG activities in other cortical regions beyond those involved in recognizing speech. In particular, we sought to determine whether cortical areas thought to be important for determining sound source distance from intensity cues contributed to processing when intensity cues were minimized and other cues were more informative.

Results from the present study show that phonetic familiarity increases the accuracy of auditory distance estimation (i.e., English and Bengali was perceived more accurately played forwards than backwards), and that although many of the same brain regions are engaged during processing of all speech-like sounds, EEG dynamics in these brain regions vary depending on a participants’ familiarity with the sounds they are hearing.

## Results

### Behavioral Results

The sound stimuli used in the current experiment were forward and backwards played versions of English and Bengali speech samples recorded at distances of 2 m or 30 m away from a speaker in an open field (see Materials and Methods section for details). Recordings were equalized in overall intensity to minimize intensity cues to distance. Fluent speakers of English with no Bengali language background (n = 15) heard these sounds played over speakers at a comfortable volume level while continuous EEG was collected from 248 channels. Participants were instructed to respond ‘Far’ or ‘Near’ by pressing keys to indicate whether they thought the pre-recorded sound source was 2 m or 30 m away after each presentation of a sound. Accuracy was measured as the percent of total trials in which the participant responded correctly. No feedback of performance was given at any point during testing.

**Figure 1:**
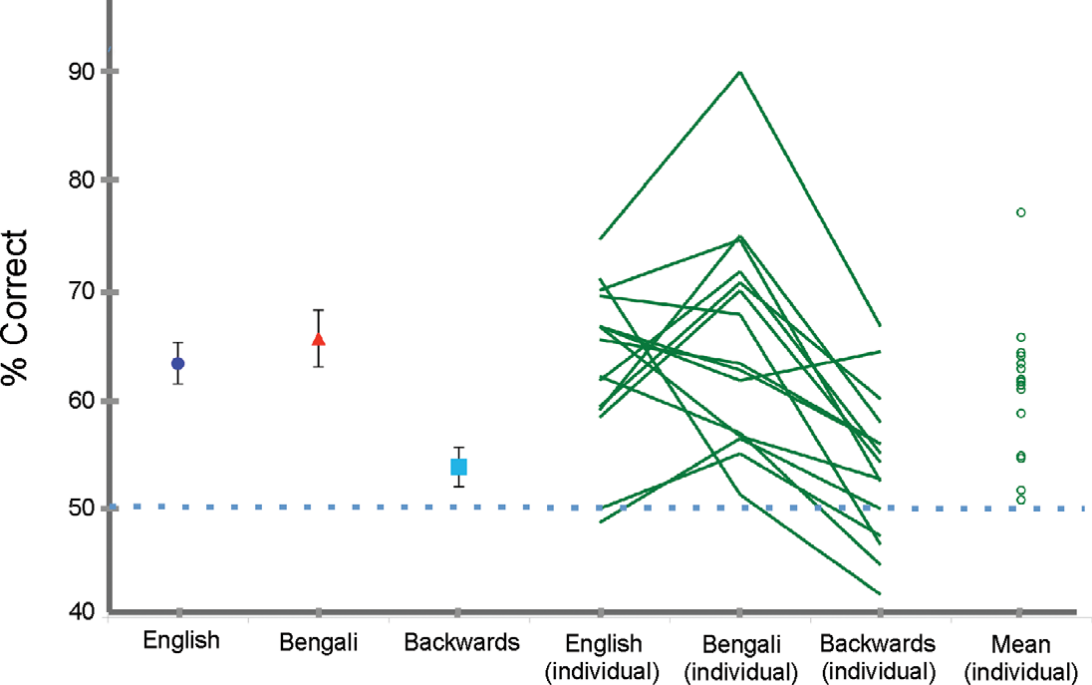
Accuracy for different speech categories. (left) Mean accuracy for each speech category. Error bars show the standard error of the mean. The dotted blue line indicates chance performance level. (right) Green lines show the performance of each participant for different speech categories and green circles indicate individuals’ mean performance across speech categories.

The mean accuracy of auditory distance judgments for participants, averaged across sound categories, was 61% correct, which was significantly above chance (t_(14)_ = 6.53, *p*<0.001, Cohen’s d = 1.67). Average accuracy scores for English, Bengali, and backward speech categories are shown in Figure 1. A single factor (speech category) repeated-measures ANOVA showed that the means for different categories of speech were significantly different, (F_(2, 28)_ = 14.77, *p*<0.001, η_p_^2^ = .77). Planned comparison paired sample t-tests revealed that the mean accuracies for English (t(14) = 4.115, p = .001, Cohen’s d = 1.337) and Bengali, (t(14) = 6.181, p<.001, Cohen’s d = 1.363) were significantly higher than for backwards speech.

There were also substantial individual differences in accuracy (see Figure 1). Across participants, mean task performance ranged from 51% to 77% correct, leaving even the best performer far away from perfect performance, and demonstrating that some participants found the task to be extremely difficult. Despite these individual differences in discrimination ability, most participants showed the same general pattern of results as in the group mean data. All but 2 of the 15 participants performed worst for the backwards speech category.

### Electrophysiological Results

Extended infomax ICA was used to separate each participant’s EEG data into ICs. Clusters of ICs were identified using a distance metric composed of principal component analysis (PCA) reduced event-related spectral perturbations (ERSPs), dipole locations, IC log power spectra, and K-means clustering (see Materials and Methods section for more detail).

**Figure 2:**
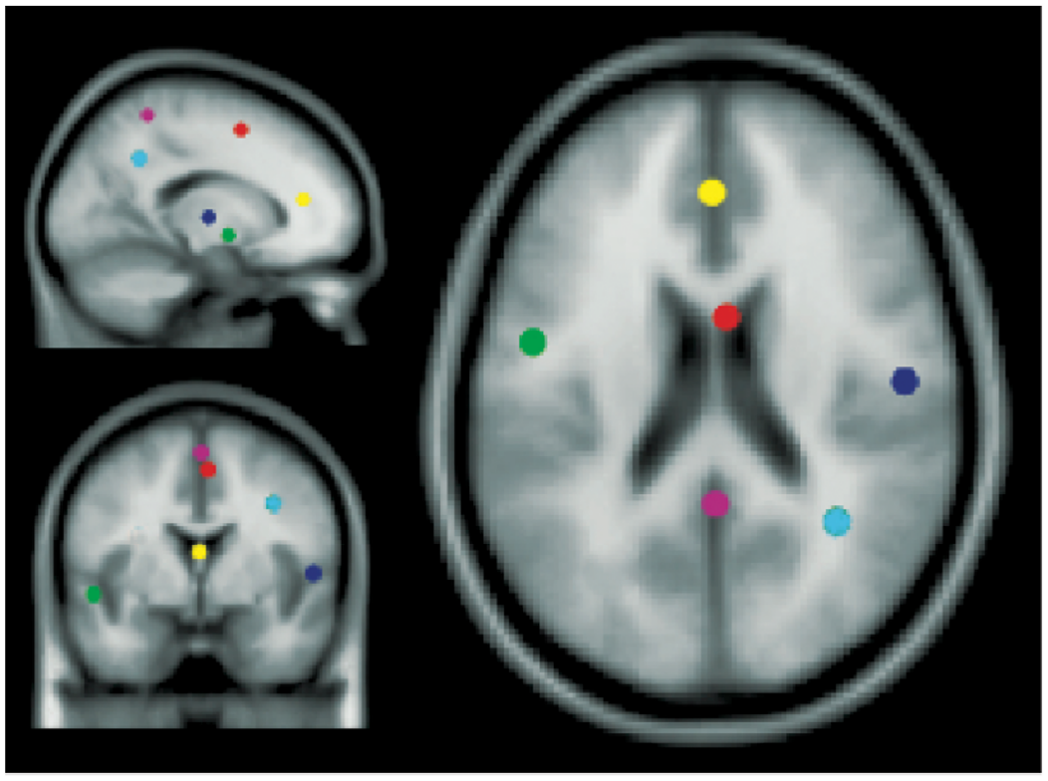
Independent component (IC) cluster centroids. Centroids of IC clusters as determined by equivalent dipole reconstructions with locations in or near: left superior temporal gyrus (green), right superior temporal gyrus (blue), anterior cingulate cortex (yellow), medial frontal gyrus (red), precuneus (pink), and right inferior parietal lobe (cyan).

ERSPs, which show mean log spectral power changes relative to baseline across trials at a range of frequencies and time latencies [17], revealed spectral dynamics of several component clusters time-locked to task events. For some clusters, these dynamics were significantly different between speech categories. We report here IC clusters centered in or near the left and right temporal lobes, as well as clusters in other non-auditory regions that showed significant differences between speech categories in their spectral dynamics and/or significant event-related changes in IC spectrum. The centroid locations of IC clusters located in or near left-temporal lobe (STG), right-temporal lobe (STG), anterior cingulate cortex (ACC), medial frontal gyrus (MFG), parietal cortex (precuneus), and the right inferior parietal lobule are shown in Figure 2. There were other clusters of ICs, but these are not described here, because processes typically associated with those clusters were not a focus of the current study.

ERSP images for all IC clusters revealed similar event-related dynamics across speech categories. For this reason, all images of ERSPs in this paper display the average ERSPs across speech conditions. In constructing the ERSPs, the temporal axes of the single-trial spectrograms were time-warped to make the number of data points from stimulus onset to response onset the same for all trials, allowing averaging and display of stimulus and response event-related phenomena in the same figure. The abscissa of the ERSP images thus shows changes in EEG spectral power as they occur through a temporal dimension normalized to the mean response time (1700 ms) rather than over absolute time. Details of this normalization procedure are provided below in the EEG analysis subsection of the Materials and Methods section.

#### Temporal IC clusters

**Figure 3:**
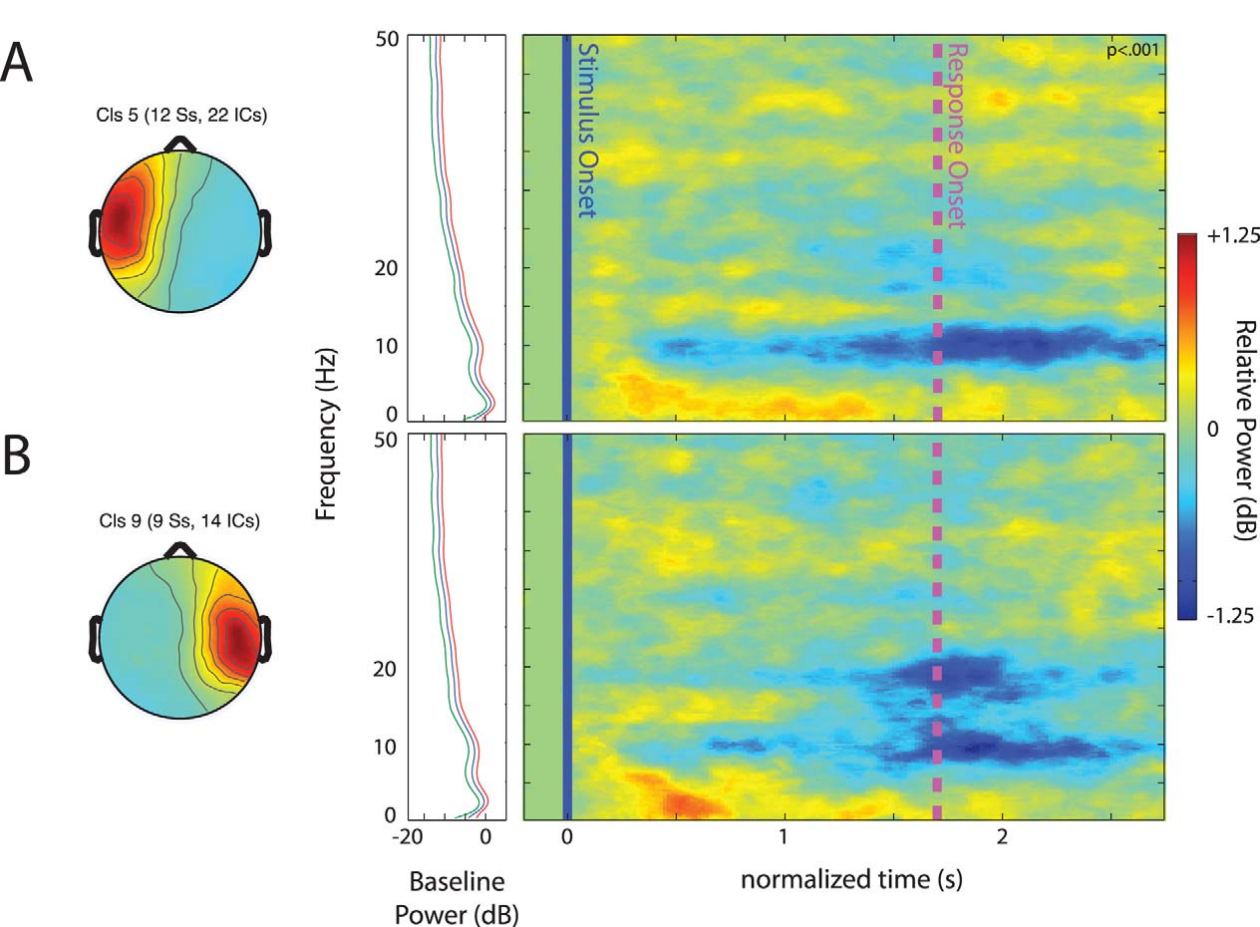
Event-related spectral perturbations (ERSPs) in temporal clusters. ERSPs and associated scalp maps for the (A) left temporal and (B) the right temporal independent component cluster. The frames immediately to the left of the ERSP images show the average baseline log power spectrum (−200–0 ms, blue trace) that was subtracted from individual component activities to generate ERSPs. The red and green traces in these frames show the average upper and lower significance threshold (p<.001) across individuals. The ERSP images on the right were created by averaging component ERSP images from individual participants after masking non-significant perturbations from baseline (p<.001). Vertical blue lines in the ERSP image indicate speech delivery onsets, and pink dashed lines indicate motor responses. Colors in the images indicate the relative log power (in dB) at that frequency and latency (normalized) relative to the power at that frequency during the baseline period.

ERSPs averaged across speech categories for the temporal IC clusters are shown in Figure 3. Changes to the IC spectrum were only analyzed within times and frequencies in which there was at least a 0.5 dB change from baseline. This method was used to define windows to be examined for differences between ERSPs for speech categories in all reported clusters.

**Figure 4:**
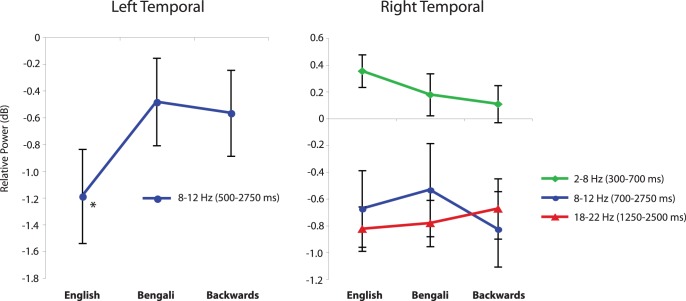
Spectral power for different speech categories in temporal clusters. Relative power in time/frequency windows that showed greater than a 0.5 dB change from baseline for the left and right temporal IC clusters, and for different speech categories. Error bars show the standard error of the mean. The asterisk indicates that the mean relative power for English speech was significantly different from that of Bengali and backwards speech.

For the left temporal cluster, the only time-frequency window that showed a larger than 0.5 dB change from baseline was 8–12 Hz between 500 ms and 2750 ms. Figure 4 shows the power, relative to baseline, in this window for different speech conditions. A repeated-measures ANOVA with speech condition as the only factor showed that there was a significant difference between means (F(2, 40) = 7.80, p = .001, η_p_^2^ = .281). Post-hoc one-sample t-tests, interpreted with Bonferroni corrections for three comparisons showed that the decrease in 8–12 Hz power was significantly larger for English than for Bengali (t(20) = 4.267, p<.001, Cohen’s d = 1.31), and Backwards speech (t(20) = 2.51, p = .021, Cohen’s d = .73). Differences between speech categories were expected considering that left temporal areas are believed to be important for the processing of familiar speech (see [16], for review). In the current case, the larger alpha band power decrease could be related to stronger engagement of left temporal areas during the processing of English speech sounds by English speakers [16], [18].

For the right temporal cluster, three different time/frequency windows showed larger than 0.5 dB changes from baseline. These are also shown in Figure 4. Relative power in these windows was compared using a (3) (window) × (3) (speech category) repeated-measures ANOVA. Only a main effect of window was found (F(2, 52) = 7.12, p = .003, η_p_^2^ = .354), as different windows contained different degrees and directions of power changes. The finding that right temporal areas are engaged by this task is consistent with previous work on intensity cues in auditory distance [12], [13], but differences in the time course of frequencies was not found between speech categories, leaving the behavioral differences unassociated with differential EEG activity of right temporal areas.

#### Frontal IC clusters

**Figure 5:**
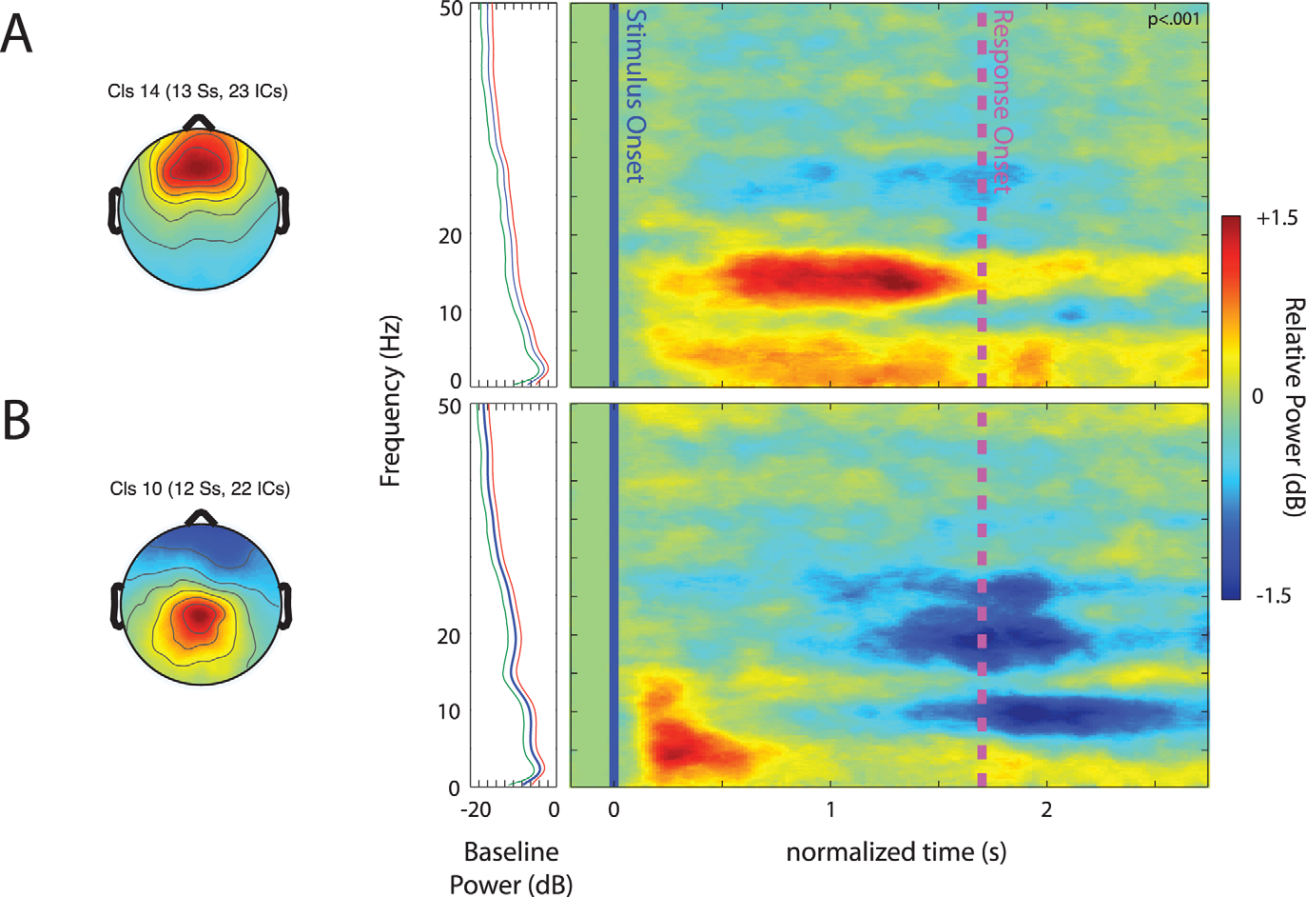
Event-related spectral perturbations (ERSPs) in frontal clusters. Scalp maps and their associated time-warped ERSP images for the ACC (A) and medial frontal gyrus (B) IC clusters. Other details as in Figure 3.

IC centroids for frontal clusters were centered in the anterior cingulate cortex (ACC) and the medial frontal gyrus (MFG) with individual ICs in or near those locations. The ERSPs associated with these clusters are shown in Figure 5.

**Figure 6:**
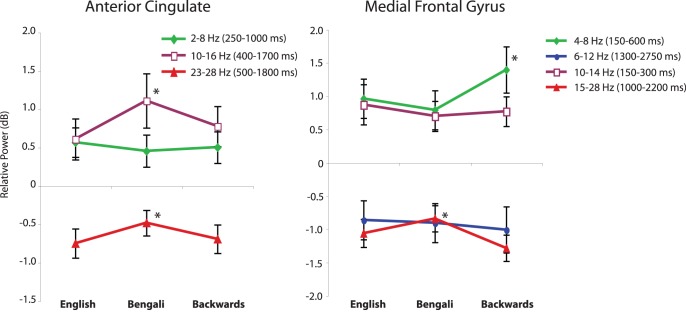
Spectral power for different speech categories in frontal clusters. Relative power in time/frequency windows for which there was more than a 0.5 dB change from baseline. Error bars show the standard error of the mean. Asterisks denote means that were significantly different.

For the ACC cluster, the mean ERSP shows a sustained response in low-frequency power (2–8 Hz), increase at high-alpha/low-beta frequencies (10–16 Hz), and a high beta-band response (23–28 Hz) beginning after speech onset and lasting until the motor response. Figure 6 plots the strength of these changes in the IC spectrum across speech categories. A (3) (window) × (3) (speech category) repeated-measures ANOVA revealed a main effect of window (F(2, 88) = 23.87, p<.001, η_p_^2^ = .520), reflecting the different directions and strength of change in different windows, and a main effect of speech category (F(2, 88) = 3.24, p = .049, η_p_^2^ = .128), showing that different categories exhibited a different mean power spectral change. The window x speech category interaction was also significant (F(4, 88) = 4.181, p = .004, η_p_^2^ = .160).

Paired-sample t-tests were performed on all possible within window comparisons between speech categories. T-tests were interpreted with Bonferroni corrections for 9 multiple comparisons. Bengali speech had significantly more 10–16 Hz power increase than either the English (t(22) = 2.82, p = .01, Cohen’s d = .93) or backwards speech categories (t(22) = 2.32, p = .03, Cohen’s d = .94). It was also the case that 23–28 Hz power spectral decreases were smaller for Bengali than for Backwards speech (t(22) = 2.30, p = .031, Cohen’s d = .73).

In the medial frontal gyrus IC cluster, the ERSP showed a significant theta (4–8 Hz) and high-alpha/low-beta (10–14 Hz) power increase shortly after stimulus onset. There were also significant decreases in 6–12 Hz power and (possibly 1^st^ harmonic) beta frequency bands (15–28 Hz) in the time surrounding response onset. Figure 6 shows the relative power for each of these frequency ranges during the times they exceeded a 0.5 dB change from baseline. A (4) (window) × (3) (speech category) ANOVA showed a main effect of window (F(3, 120) = 25.47, p<.001, η_p_^2^ = .560) as well as a significant window × speech category interaction (F(6, 120) = 2.40, p = .032, η_p_^2^ = .107).

To explore the sources of the interaction, four separate repeated measures ANOVAs were conducted on each window with speech category as the sole factor. Means for speech categories were significantly different for the theta band power difference (4–8 Hz) (F(2, 40) = 4.05, p = .025, η_p_^2^ = .168), and for the beta band power difference (15–28 Hz) (F(2, 40) = 4.81, p = .013, η_p_^2^ = .194). Paired sample t-tests, interpreted with Bonferroni corrections for 6 comparisons, showed that following Bengali speech there was significantly less event-related change in (4–8 Hz) theta power (t(20) = 2.98, p = .007, Cohen’s d = 1.04) and in 15–28 Hz beta (t(20) = 3.44, p = .003, Cohen’s d = 1.09) than backwards speech. The same trend was apparent in the comparison of English and backwards speech for both theta power (t(20) = 1.73, p = .10, Cohen’s d = .56) and for beta power (t(20) = 1.77, p = .092, Cohen’s d = .55), but contrasts were only marginally significant.

#### Parietal IC clusters

**Figure 7:**
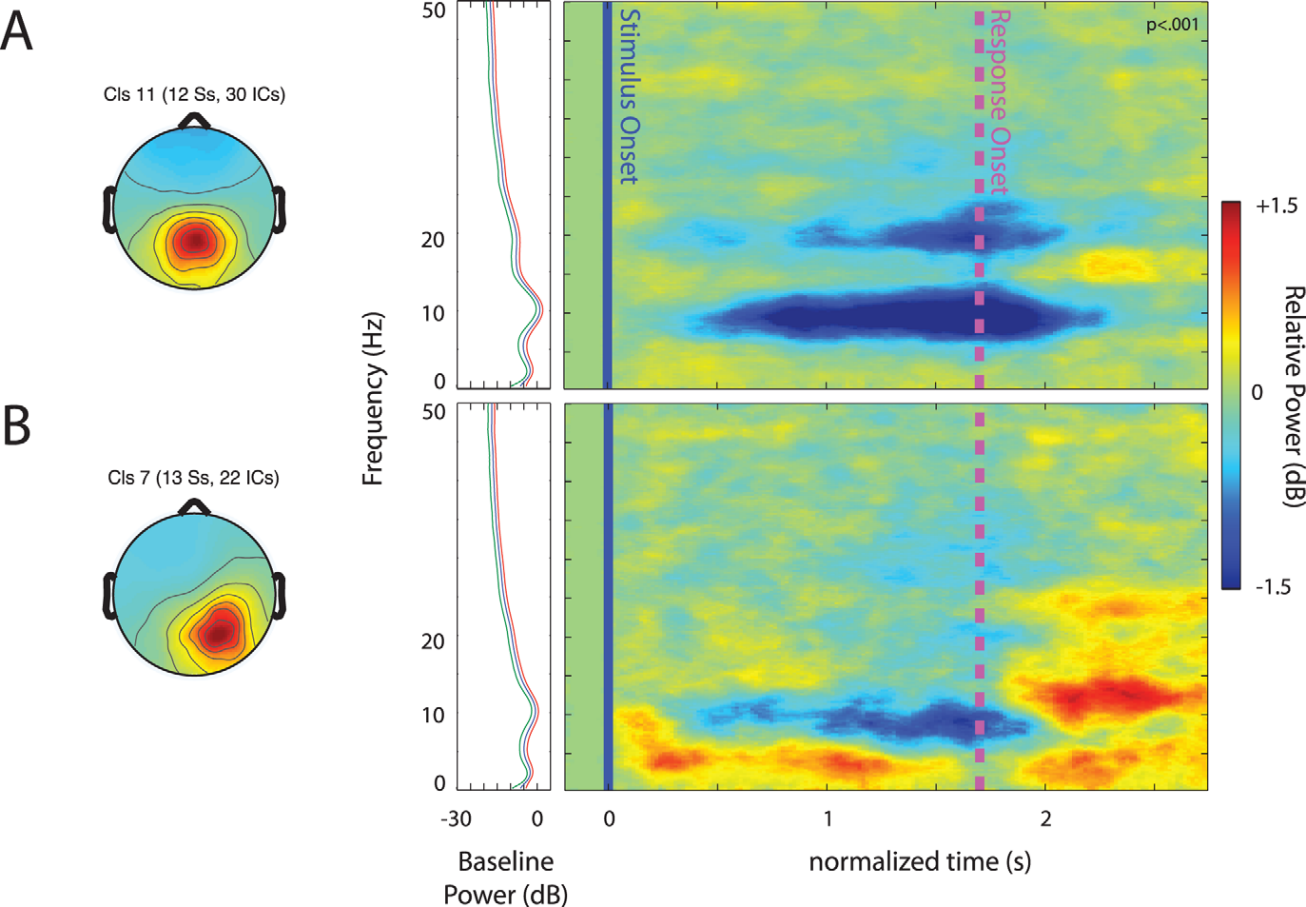
Event-related spectral perturbations (ERSPs) in parietal clusters. Scalp maps and ERSPs associated with clusters of ICs centered in precuneus (A) and the right inferior parietal lobule (B). Other details as in Figure 3.

Two parietal clusters of ICs showed task related spectral perturbations. Figure 7 shows the scalp maps and ERSPs of a cluster centered in or around precuneus (A) and another cluster centered in or near the right inferior parietal lobule (B).

**Figure 8:**
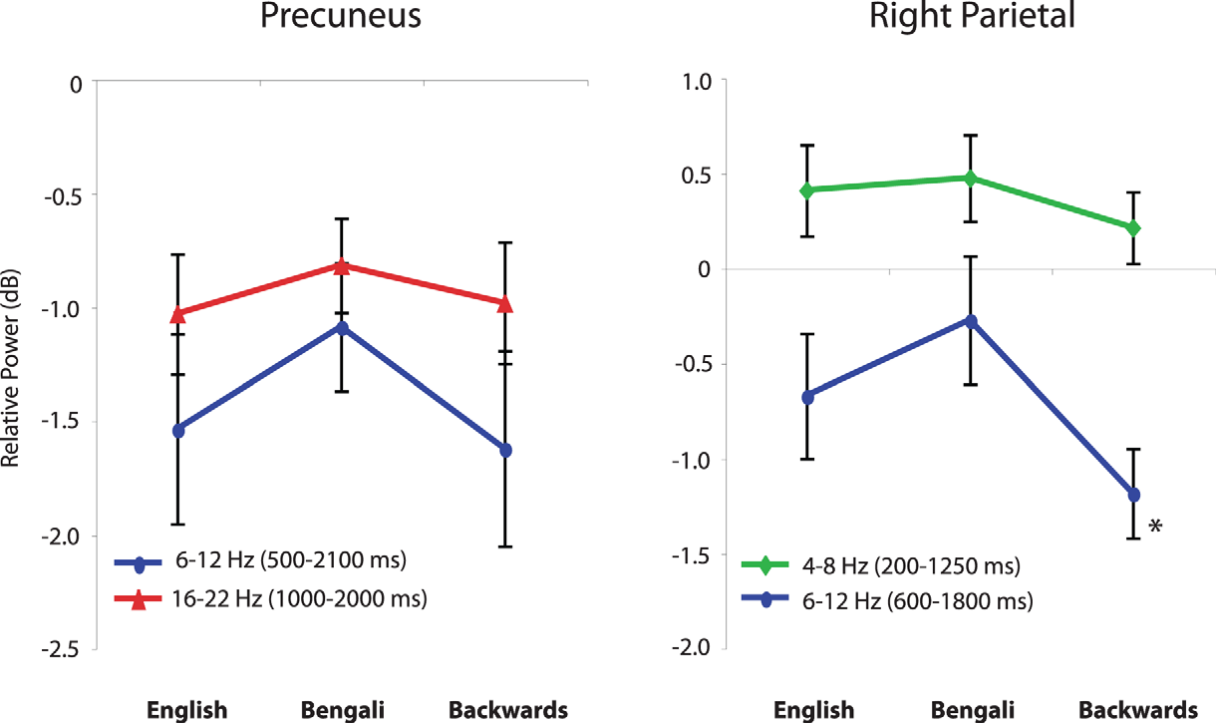
Spectral power for different speech categories in parietal clusters. Relative power for theta, alpha, and high-beta bands centered in the precuneus and right inferior parietal lobule. Error bars show the standard error of the mean. The asterisk indicates that the mean relative power for backwards speech was significantly different from that of Bengali speech.

The IC cluster centered in the precuneus of the parietal lobe showed a significant and sustained decrease in high-theta/alpha band 6–12 Hz power and high beta (18–30 Hz) power beginning approximately 500 ms after the stimulus onset and dissipating following the manual response. Figure 8 shows the relative power at these frequencies across speech categories and the normalized time frames within which they were measured. A (2) (window) x (3) (speech category) repeated-measures ANOVA found only a marginally significant main effect of window (F(1, 58) = 3.78, p = .062, η_p_^2^ = .115), owing to the stronger decrease in 6–12 Hz, than in beta band power.

For the right parietal cluster, there was a sustained theta (4–8 Hz) response starting shortly after the onset of speech and continuing until the response. There was also an alpha power (8–12 Hz) decrease between about 600 ms and 1800 ms. After the response onset, at several frequencies, there was a large power increase; these changes were not analyzed further since they occurred within the inter-trial interval. A (2) (window) × (3) (category) repeated-measures ANOVA revealed a main effect of window (F(1, 44) = 25.00, p<.001, η_p_^2^ = .532), demonstrating differences in extent and of direction of relative theta and alpha power. There was also a significant main effect of speech category (F(2, 44) = 3.99, p = .038, η_p_^2^ = .138) and a marginally significant window x speech category interaction (F(2, 44) = 2.69, p = .079, η_p_^2^ = .109). To get a better idea of the factors contributing to these effects, we conducted paired-sample t-tests on each possible comparison of speech category means within the same window. The only statistically significant effect was a stronger decrease in alpha band 8–12 Hz power following backwards compared to Bengali speech (t(22) = 3.97, p = .001, Cohen’s d = 1.29).

## Discussion

In line with previous studies on auditory distance perception in humans, we found that participants’ judgments of source distance for intensity-normalized speech sounds were generally poor. Participants distinguished intensity-normalized speech pre-recorded at a distance of either 2 or 30 m with a mean accuracy of only 61% correct. They judged the distance of forward speech more accurately than backwards speech, replicating several prior reports that people are better at estimating the source distance of familiar speech [3], [6], [10]. The current study extends past behavioral studies by showing that such benefits reflect the processing of familiar phonemes or simpler acoustic features rather than lexical familiarity, because English speakers were as accurate at distinguishing source distance when the speech being judged was in an unfamiliar language (Bengali). This finding also suggests that novelty per se cannot fully account for the differences in ranging accuracy between natural and reversed speech, since the novelty of the unfamiliar language did not degrade performance.

Why it is easier to judge the distance of a speaker producing natural speech remains unclear. Brungart and Scott [3] suggested that time-reversed speech contains all of the relevant acoustic cues for judging the distance of a speaker producing speech at conversational levels, but that phonetic information might be necessary for a listener to correctly interpret these cues when speech was produced at higher amplitudes. In that study, distant-dependent variations in intensity level were preserved and likely contributed to participants’ performance. McGregor and colleagues [10] equalized the loudness of speech stimuli in their experiment, and found that reversing speech degraded performance (as was seen in the current study). These findings suggest that phonetic information may be particularly relevant when intensity cues are not reliable indicators of source distance. This leaves the question of why phonetic processing might increase the availability of localization cues unanswered. One possibility is that familiar speech is processed more automatically, freeing brain resources for extracting auditory distance cues. Another possible factor, not considered in previous work, is that natural speech sounds are not only more familiar, they are also more reproducible. If reproducible sounds activate motor representations relevant to producing those sounds, then the availability of multimodal stimulus representations could enhance processing of acoustic cues [20], [21]. This explanation predicts that the more easily imitated a sound is, the better individuals should be able to judge how far it has traveled.

Past neuroimaging work has implicated right temporal brain areas in auditory distance perception [8], [11]–[13]. Here we observed an IC process cluster centered in the right superior temporal gyrus that exhibited stimulus-related changes in EEG activity during performance of the distance judgment task. These changes were comparable across different speech conditions, however, and thus cannot account for the observed behavioral differences. For a similar IC cluster in the left temporal lobe, listening to English speech sounds produced more alpha power decrease (‘alpha blocking’) than other speech categories, but since distance perception with English and Bengali speech was comparable, this difference also cannot account for the behavioral results.

Recent observations of brain activity changes produced during listening to backward speech using fMRI [22] and MEG [23] imaging suggest that auditory-specialized networks in the temporal lobes respond as reliably and systematically to backwards speech as to natural speech, consistent with the current findings. However, it remains possible that differences in processing in these regions not indexed by the IC source EEG power-change measures used in this study (e.g., fine-scale differences in the spatial distribution of neural activity or in auditory receptive fields) might significantly contribute to differences in accuracy across conditions.

We also observed power decreases at alpha and high-beta band EEG frequencies in an IC cluster located in or near precuneus. Studies of auditory spatial processing have implicated the parietal lobes in spatial attention [24], [25]. For instance, in a task where participants had to attend to either the left or right side of auditory space to detect a target sound, fMRI data showed that there was stronger activation in the left and right precuneus of the superior parietal lobes in a task where targets on the left and right side of auditory space were not present [25]. The activity near the precuneus centered IC cluster in this study also suggests that estimating source distance may involve the superior parietal lobe, as it does for localization in horizontal space. However, we did not manipulate attention in the current task and thus cannot say for certain whether our finding reflects spatial attention. As with the right temporal IC cluster, the event-related spectral dynamics of the precuneus cluster processes were not significantly different between speech categories, providing no information concerning brain-substrates underlying behavioral differences.

An IC cluster centered in or near the anterior cingulate cortex (ACC) showed significant perturbations in low frequency, low-beta, and high-beta band EEG power between the onset of speech and response. Onton et al. [19] showed that the level of relative EEG power in theta and low-beta bands for a similar cluster of ICs was related to the number of items stored in working memory. In that study, larger power was produced by the IC cluster when more items were being stored, suggesting that larger relative power was indicative of more effortful maintenance of memories. Others have implicated the ACC in attention associated with selective processing of relevant parts of an auditory scene [26].

In the current study, we saw the largest relative power changes for Bengali speech items in the low and high beta ranges for an IC cluster near ACC. One possible reason for this finding may be that participants were trying to maintain and/or attend to information related to making the distance judgment. Distance cues in English speech may be more salient and easier to maintain because of their lexical and phonetic familiarity [27], and may thus require less engagement of ACC (i.e., greater ACC activity is required to effectively process Bengali speech). Backwards speech may also show less engagement of ACC because information cannot be adequately maintained in working memory (backwards speech is harder to reproduce, and thus rehearse), and because selective attention may be less effective when lexical or phonetic familiarity cues are not contributing to auditory scene analysis. The current study was not designed to assess the validity of such a hypothesis. However, the observed differences in the ACC suggest that future research on familiarity effects and auditory distance perception should further investigate how working memory and attentional processing vary between different categories of stimuli.

For an IC cluster centered in or near the medial frontal gyrus, there was a relative increase in the theta band power for all conditions. This increase closely followed stimulus onsets, and was largest for backwards speech. Another IC cluster associated with the right inferior parietal region showed relative decreases in the alpha band. This decrease appeared to be more closely linked with the response onset than with the stimulus onset and was again largest for backwards speech. Several neuroimaging studies have implicated parietal-frontal networks in sound localization, and have identified the right parietal cortex as being particularly important for higher-order spatial processes (reviewed by [28]). It has also been suggested that circuits in the medial frontal gyrus are specialized for gathering information in perceptual classification tasks [29]. In general, the current results are consistent with the idea that extracting spatial cues from natural speech requires less engagement of fronto-parietal circuits than is the case for backwards speech. However, there is another possible reason why we may have seen stronger responses in the backwards speech condition. Medial frontal and right parietal areas have also been implicated in processing events that are novel [30]. Backwards speech was the most novel speech category used here. It therefore could also be the case that medial frontal and right parietal areas are more active for backwards speech because they are working to process both novelty and auditory spatial information. This hypothesis might also explain why performance was worst for the backwards speech category. Even though source locations may be automatically assessed for novel stimuli [31], overlap in the brain regions involved in novelty processing and auditory spatial perception might actually hurt performance. In this case, distance estimation may have been worse for novel backwards speech because the resources available to process spatial information were more limited. Either way, the current findings are the first indication that the coherence of particular EEG frequency components in these non-auditory regions may relate to differences in the accuracy of auditory distance perception. This relationship may be similar to that observed in auditory cortex between theta-band phase patterns and speech intelligibility [32].

The temporal dynamics of changes in power across different frequency bands revealed in ERSPs during task performance (Figures 3, 5, & 7) suggest that a complex, widespread, and well-coordinated bout of brain activity occurs during performance of this auditory task. Surprisingly, the earliest stimulus-related changes are most evident in the cluster associated with the medial frontal gyrus and involve increases in EEG power, whereas other components showed later onset of power changes that involved decreases in power (e.g., in the alpha band). It is not clear whether these dynamics contribute to perceptual acuity or performance. This could potentially be revealed in future studies by analyzing individual differences in accuracy and brain dynamics.

The current study represents a first attempt at identifying changes in brain activities that underlie judgments of auditory distance, and is thus limited in several respects. We did not directly measure participants’ ability to localize sound sources and so cannot assess whether their performance in this dichotomous auditory task accurately reflects their spatial acuity. Furthermore, it is possible that participants’ used acoustic cues to differentiate sounds without perceiving them as spatial cues (i.e., they could distinguish the sounds, but did not perceive the differences as corresponding to changes in position of the source). Given that the recordings of playbacks were of the same speech-like sounds broadcast from different distances, it seems likely that most or all of the differences between recordings would reflect propagation-related cues. Also, given that sound localization often occurs rapidly and involuntarily, participants’ brains are likely continuously monitoring for the presence of such cues. Nevertheless, additional studies will be needed to definitively identify the neural substrates of auditory distance estimation, as well as the factors that constrain the accuracy with which a particular individual can judge the distance to a sound source.

## Materials and Methods

### Ethics Statement

The study was approved by the Human Research Protections Program of the University of California, San Diego. All participants were asked to read and sign an informed consent form before participating in the study.

### Participants and EEG Data Acquisition

Seventeen participants from the University at California, San Diego, were paid to take part in the study. All participants had normal hearing. All were fluent speakers of English with no Bengali language background. Two participants were dropped from analyses because of errors that occurred in the collection of EEG data.

EEG was recorded from 248 channels at a sampling rate of 512 Hz, 24-bit A/D resolution, referenced to the CMS-DRL ground using a Biosemi ActiveTwo system (Biosemi, Netherlands). Caps with a custom whole-head montage positioned electrode wells over most of the skull, forehead, and lateral face surface. The wells were filled with water based conductive gel and the electrodes were placed within them. The locations of electrodes, relative to skull landmarks were recorded for each participant (Polhemus, Inc). Input impedances for electrodes were brought below 20 kΩ before data collection. If the impedance criterion was not reached for an electrode, that electrode was rejected from analyses. Stimulus and response onsets were recorded in a separate event channel.

### Stimuli and Experimental Procedures

Three categories of sound were used in order to investigate effects of familiarity on the perception of auditory distance and EEG dynamics. These categories were lexically and phonetically familiar (English speech), phonetically familiar (Bengali speech), and unfamiliar (backwards speech). The same speaker, who was fluent in English and Bengali, produced all speech sounds in a natural manner. These particular categories of stimuli were chosen and the same speaker was used so that the basic spectral structure would be very similar across categories [33], [34]. Because backwards speech has identical spectral characteristics and temporal dynamics as forward speech, it is the closest to a ‘non-speech’ stimulus that one can get while still being very physically similar to natural speech. English speech consisted of the following phrases/words: “Don’t ask me to carry an oily rag like that”, “Threat”, “Warning”, “Emergency”, “How far away do you think I am?”, “Look out”, “Over here”, “Caution”, “Hello”, and “Goodbye”. Bengali phrases were: “Amaka ooghta tooltaa bolo-nah”, “Aa kha nae”, “Aloo”, “Kawla”, “Choo noo dau”, “Shaub dhan ah”, “Aamee kau tou dor ah ache?”, “Mo mosh kar”, “Hah”, and “Nah.” With the exception of hello and goodbye, all English words and sequences were chosen in order to replicate previous work [3], [10]. Backwards speech was created from both English and Bengali speech (“Don’t ask me to carry an oily rag like that”, “Threat”, “Warning”, “Emergency”, “How far away do you think I am?”, “Look out”, “Goodbye”, “Aa kha nae”, “Choo noo dau”, and “Aamee kau tou dor ah ache?”). Pilot studies revealed no differences between backwards speech made from English and Bengali stimuli. In order to make neuroimaging comparisons more easily interpretable, we collapsed across backwards English and Bengali stimuli in our design and analysis.

All categories of speech were recorded in a single recording session. Sounds were initially recorded in the same room using an AKG D9000 microphone (frequency range 20 Hz –20 kHz) approximately 6 inches from the talker’s mouth (70–90 dB SPL peak) and a digital recorder (Sony MD Walkman Mz-NH900, recording in.wav format). Backward sounds were created by reversing waveforms using the acoustic software program Peak (BIAS, Inc.). All sounds were broadcast from a SUNN speaker (model 1201, Fender Musical Instruments Corporation) into an open grass field at night in order to minimize environmental noise. The free field environment was chosen to replicate the recording/testing methods of previous work [3], [10]. Broadcasts were recorded from a distance of 2 m (Near) and 30 m (Far), using the same setup as the initial recordings. Thus, there were 20 recordings for each category of sound, 10 of which were recorded from Near and 10 of which were Far, giving a total of 60 sounds. All the sounds were then normalized to a constant intensity level (−10 dB FS) using PEAK to minimize the availability of intensity as a distance cue.

Participants were seated in a dimly lit room in front of a computer screen and keyboard. Instructions and feedback after responses were presented via the computer screen using ERICA software [35]. Sounds were presented using the same software over two speakers approximately three feet in front of participants. Sound levels (peak level <75 dB SPL), room, and speaker arrangement were the same for all participants. Pilot studies showed that the same general pattern of behavioral results was obtained using headphones. Because of the pilot studies, and previous studies showing similar results [3], [10], we suspect that room characteristics (e.g., reverberation, noise, etc) did not significantly affect the results of the current study.

Participants were told that they would be presented with sounds that were recorded at near or far distances and that their task throughout the entire experiment was to use their right hand to hit the ‘J’ key on the computer keyboard if the sound was Near and the ‘L’ key if the sound was Far. They were also told that sounds were altered such that intensity was not a viable discrimination cue.

There were 3 blocks of 60 stimulus presentations. Self-paced breaks were taken in between blocks, none of which lasted longer than five minutes. Stimuli from the different speech categories, recorded at both Near and Far distances, were presented in random order. Participants were given 4 seconds after the onset of a sound to respond. No feedback was given throughout testing. If no response was made on a given trial, the computer screen displayed the request “Please make a response next time” before moving on to the next trial. These trials were removed from analysis.

### EEG Analysis

Data was analyzed using the open source EEGLAB toolbox for Matlab ([36]; http://sccn.ucsd.edu/eeglab). Raw EEG data was visually inspected for high-amplitude, high-frequency muscle noise and other artifacts. Segments of EEG that contained these artifacts were removed from analysis. Eye movements were not a criterion for removal. Data from electrodes identified as having poor skin conductance by their abnormal activity patterns was also rejected. After this selection process, data from 134–224 electrodes (M = 186, SD = 32) remained for each participant. EEG data was then digitally high pass filtered with a cutoff of 1 Hz, re-sampled at 250 Hz, and re-referenced to the average of the retained electrodes.

Each participant’s filtered EEG data was entered into a full-rank extended infomax ICA using the *binica()* function [37] of the EEGLAB toolbox. See Jung et al. [38] for derivation of the infomax algorithm; for the application of the algorithm to EEG data see Makeig et al. [14] and Onton and Makeig [15]. Decompositions used default extended-mode *binica()* training parameters. Extended infomax ICA allows the recovery of components with supra- or sub-Gaussian activity distributions. For instance, 60-Hz line noise has a supra Gaussian activity distribution which in favorable circumstances can be separated from other data by using extended infomax ICA.

The scalp topographies, time courses, and spectra of ICs were visually inspected to separate brain activity from non-brain artifacts (e.g., muscle noise, line noise). For instance, ICs with spectra that showed high power in the high frequencies were rejected for being muscle artifacts (for details on IC rejection see [15]). An equivalent current dipole for each IC was then computed by using a boundary element head model co-registered to each participant’s electrode locations by warping the electrode locations to the model head using tools from the EEGLAB *dipfit()* plug-in. If the best fitting equivalent-dipole had more than 15% residual variance over all electrodes from the IC scalp map, the component was rejected from further analysis. ICs with an equivalent-dipole outside of the brain were also rejected. The mean number of remaining ICs per subject was 19 (SD = 7; range, 9–32).

Continuous EEG was split into 4-s epochs beginning 1 second before stimulus onset and ending 3 seconds after it. ERSP transforms were computed for brain ICs to examine processing-related changes in the EEG power spectrum. ERSPs plot event-related changes in spectral power from baseline across a wide range of frequencies [17]. The power spectrum in time windows centered in the 200 ms prior to stimulus onsets was used as the mean baseline. Default EEGLAB Fast-Fourier transform (FFT) parameters of the EEGLAB *newtimef()* function were used to estimate spectral power in overlapping time/frequency windows. The single-trial spectrograms were linearly time-warped so as to produce equal numbers of data points between stimulus onset and the key press in all trials. Therefore, ERSP results between the labeled stimulus and response times reflect mean spectral perturbations proportionately following the stimulus and before the motor response.

The identification of clusters of similar ICs within and between participants was based on the similarities of their scalp map topographies, IC equivalent-dipole locations, log power spectra, and ERSPs. Pre-clustering involved dimension reduction using PCA to obtain an IC pairwise distance metric. K-means was then used to cluster components into 12 separate clusters, one of which was ignored as it contained ICs that were at least 3 standard deviations away from fitting into any of the other clusters (as per the clustering metric).
